# Porous PLAs with Controllable Density by FDM 3D Printing and Chemical Foaming Agent

**DOI:** 10.3390/mi12080866

**Published:** 2021-07-23

**Authors:** A. R. Damanpack, André Sousa, M. Bodaghi

**Affiliations:** 1 Department of Mechanical and Electrical Engineering, University of Southern Denmark, DK-5230 Odense, Denmark; anran@sdu.dk; 2 Department of Engineering, School of Science and Technology, Nottingham Trent University, Nottingham NG11 8NS, UK; mahdi.bodaghi@ntu.ac.uk

**Keywords:** lightweight foams, porous materials, chemical blowing agents, 3D printing, FDM, closed-form solutions

## Abstract

This paper shows how fused decomposition modeling (FDM), as a three-dimensional (3D) printing technology, can engineer lightweight porous foams with controllable density. The tactic is based on the 3D printing of Poly Lactic Acid filaments with a chemical blowing agent, as well as experiments to explore how FDM parameters can control material density. Foam porosity is investigated in terms of fabrication parameters such as printing temperature and flow rate, which affect the size of bubbles produced during the layer-by-layer fabrication process. It is experimentally shown that printing temperature and flow rate have significant effects on the bubbles’ size, micro-scale material connections, stiffness and strength. An analytical equation is introduced to accurately simulate the experimental results on flow rate, density, and mechanical properties in terms of printing temperature. Due to the absence of a similar concept, mathematical model and results in the specialized literature, this paper is likely to advance the state-of-the-art lightweight foams with controllable porosity and density fabricated by FDM 3D printing technology.

## 1. Introduction

Slicing software packages like Cura or PrusaSlicer allow for Computer Aided Designs to be expediently brought into numerical control code and enhance the reliability of printing hardware [1] and economies of scale [2]. Coupled with the popularization of “easy to print” thermoplastics like Poly Lactic Acid (PLA) [3], Fused Deposit Modeling (FDM) has become one of the most popular methods for rapid prototyping. FDM is a cheap and accessible 3D printing technology and perfect for beginners to 3D printing. FEM is simple to use, and 3D printers are very user-friendly. However, there are also some disadvantages to FDM. The print quality of FDM is not as good as, for instance, stereolithography or selective laser sintering. FDM is quite slow and unusable in some industries, when large numbers of parts are required quickly. The layer-by-layer fabrication in FDM can sometimes lead to problems with warping and minor shrinking.

For high-performance structures, stressed-skin designs are a proven practice in the aviation industry [4], among others. However, the issue of skin buckling arises in a structure solely composed of skins. To address this issue, spars and ribs are used in wings, but these increase the number of parts and the complexity of assembly. An alternative to ribs and spars is the usage of cores made from foams or honeycomb structures to create sandwich constructions. The advantages of FDM cores were studied in [5,6]. The usage of this FDM enables very complex geometries and the integration of multiple components in a single part. In [5], a method was proposed in which a FDM Ultem core was laid-up while wet. This method was adapted for PLA using a low-temperature curing resin [6], opening the door to the usage of core materials more sensitive to temperature. This raises the following research objective: a process optimization for a low-density FDM core.

Process optimization for FDM printing has been investigated by multiple scholars for different base materials and additives [7]. Research into mineral additives (Fe, Cu, Al and Al2O3, TIO2 hydroxyapatite), organic fillers (rice straw, wood flour) and inorganic filers (carbon fibers [8] and glass fibers), between others, is contributing to the improvement in processing with these novel feedstock materials.

For low-density materials, in recent years, interest in porous polymeric structures in FDM has also been growing [9]. These structures may present improved mechanical, thermal and physical properties [10,11]; therefore, further research on them is very pertinent. One category of porous polymeric structures is syntactic foams, which use hollow spheres in their matrix. Syntactic foams have been in use since the 1950s, and have thus been the focus of extensive research [12,13,14,15].

Syntactic foams have shown a superior performance for use in FDM compared to neat materials [16,17], and thus are very promising materials, with extensive applications. Glass-based syntactic foams also have disadvantages. They are prone to damage when exposed to large strains [18,19], stiffer and more brittle, and, although recycling glass-based synthetic foams has been suggested as possible [17], their composite nature raises questions of practicality in larger-scale recycling.

Alternatives to syntactic foams have centered around the use of gas, generally CO_2_ or nitrogen, to create porous polymeric materials. Supercritical foaming effects, for example, have been studied extensively [20,21,22,23]. In this method, using a partial gas saturation technique, a non-equilibrium gas concentration is obtained in thermoplastic polymers. This has been translated into FDM manufacturing methods, as detailed in [24]. However, all the above-mentioned processes above are considered stepwise and complicated by the same author. An alternative to supercritical foaming, with chemical blowing agents (CFAs), is already used in automotive applications [25]. These parts reduce weight compared to non-foamed polymers, improve sound and thermal insulation, have high production efficiency due to their faster cycle times, and reduce machine energy and lower costs, as there is less material consumption [26]. Emerging research in CFAs proposes the FDM printing of porous scaffolds for medical applications, but fails to present the manufacture of complex geometries [24].

This paper aims to demonstrate an approach to manufacturing lightweight PLA foams by FDM 3D-printing technology, integrated with CFAs. Two parameters, printing temperature and flow rate, are assumed to be effective parameters that may influence material tailoring [27,28] and foam density through the size of bubbles produced during fabrication. Experiments are conducted to examine the effects of printing temperature and flow rate on the bubble size, micro-scale material connections, tensile stiffness and strength. An analytical closed-form solution is developed to accurately predict the experimental data on flow rate, density and mechanical properties in terms of printing temperature. This research is likely to advance the state-of-the-art 3D- and 4D printing and unlock further potential in the design and development of lightweight foams, especially for sandwich core applications, as well as leveraging the known biodegradability of PLA [29] for recyclable, greener structures.

## 2. Concepts and Methodology

A commercially available material was used, produced by ColorFabb, under the name LW-PLA. The material has an endothermic blowing agent, with a decomposition range beginning at approximately 215 °C and a maximum gas yield at processing temperatures of 220–250 °C, matching the PLA processing temperature. The range of temperatures was thus chosen to be between 215 °C and 250 °C. An Ultimaker S5 printer was used for the FDM of the foam, with a 0.4 mm nozzle. A printing speed of 100 mm/s, layer thickness of 0.35 mm and an extrusion line width of 0.35 mm were fixed for all processes in this research. The printing bed temperature was set to 60 °C and the cooling fan’s speed was turned to maximum power. Samples of 50 × 50 mm^2^ were manufactured, with 2 layers at 0 and 90° orientation, along with ranging printing temperature and flow rates. The evaluated printing temperatures were 215, 220, 225, 250 °C. The printed samples were inspected microscopically with an inverted microscope Axio Vert.A1 FL, then photographed.

**Figure 1:**
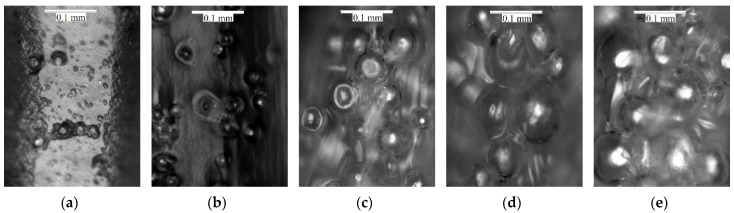
Microscopic images of 3D printed samples in different printing temperature: (**a**) 215 °C, (**b**) 220 °C, (**c**) 225 °C, (**d**) 230 °C, (**e**) 250 °C.

Figure 1 displays a typical micrograph of the samples, showing the distribution and sizing of bubbles in a random extrusion line section. As can be observed, by increasing the printing temperature and activating the foaming agent, the size and quantity of bubbles increase. This translates into a volume increase and, therefore, a reduction in material density.

As the material density is related to the printing temperature, to obtain excellent adhesion and connection between printing lies, the flow rate should be adjusted depending on printing temperature.

**Figure 2:**
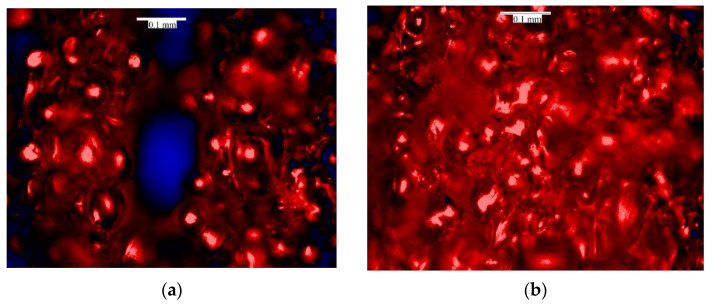
Microscopic images of 3D-printed samples in 250 °C for (**a**) 25%, (**b**) 35% infill flow rates.

To set an adequate flow rate for each printing temperature, multiple samples were fabricated, with a range of flow rates. The samples were marked and microscopically observed. The flow rate was set as the lowest value when the printing line connection was attained. In Figure 2, the effects of different flow rates are shown for a printing temperature of 250 °C. As shown in Figure 2a, a flow rate of less than 25% leads the disconnection and discontinuity of the 3D-printed lines, which is known as their being under extrusion. Therefore, according to the microscopic observations, the flow rate for the given temperature was chosen to be 35% (see Figure 2b).

**Figure 3:**
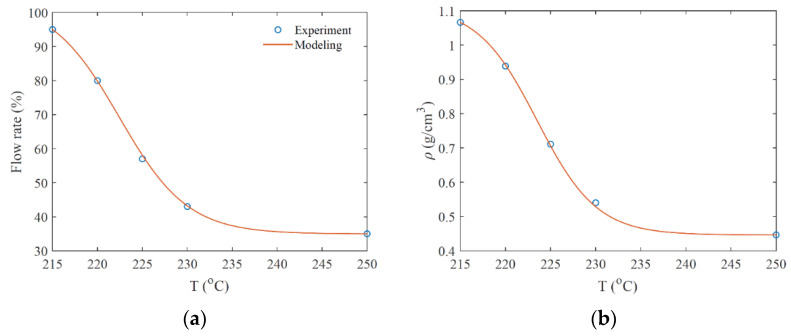
Corresponding adequate flow rate (**a**) and density (**b**) for different printing temperatures.

Similarly, to attain adhesion between printing lines, flow rates are chosen for other printing temperatures, with details displayed in Figure 3, based on further microscopical observations. As shown in Figure 3, the flow rate drops from 95% to 35%, corresponding to a 63% reduction in the flow rate. As expected, the density decreases from 1.07 g/cm^3^ to 0.44 g/cm^3^, which corresponds to a 59% reduction in density from the un-foamed polymer.

The interpolation of the experimental material properties, P(T), can be formulated as:

(1) $$P\left(T\right)=P_{h}+\left(P_{l}-P_{h}\right)\phi \left(T\right)$$

where $P_{l}$ and $P_{h}$ are the parameters at low and high temperatures, respectively. The interpolation function of ϕ(T) in terms of temperature is also set by

(2) $$\phi \left(T\right)=\frac{tanh\left(\gamma_{1}T_{g}-\gamma_{2}T\right)-tanh\left(\gamma_{1}T_{g}-\gamma_{2}T_{h}\right)}{tanh\left(\gamma_{1}T_{g}-\gamma_{2}T_{h}\right)-tanh\left(\gamma_{1}T_{g}-\gamma_{2}T_{l}\right)}$$

in which $T_{l}$, $T_{h}$, and $T_{g}$ are minimum, maximum, and critical temperatures, respectively, chosen as 215, 250 and 225 °C. $\gamma_{1}$ and $\gamma_{2}$ are the constant parameters, which are defined according to experimental data. The details of constant paxrameters are shown in Table 1 for flow rate, density and other mechanical properties.

## 3. Material Testing and Results

**Figure 4:**
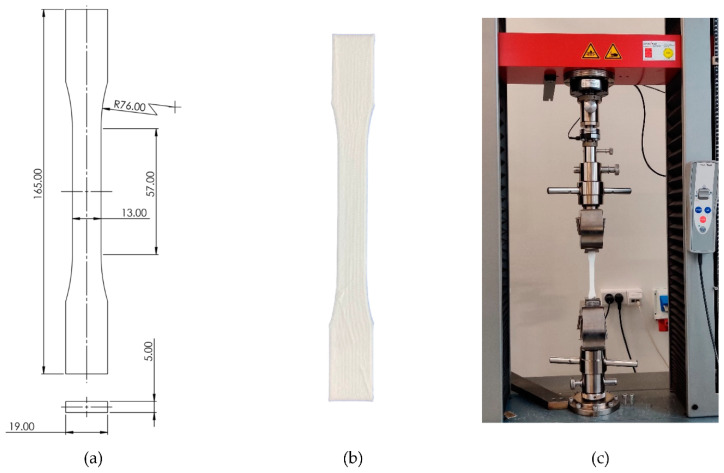
Uniaxial tensile test of PLA printed samples: (**a**) ASTM D638 dog-bone, (**b**) 3D printed dog-bone, (**c**) tensile testing machine.

Upon establishment of the flow rate/temperature curve, dog-bone specimens in accordance with ASTM D638, as shown in Figure 4, were 3D-printed. For these specimens, infill assumes a density of 100%, with a linear printing pattern oriented at −45 and 45°. Two lines form an outer shell.

**Figure 5:**
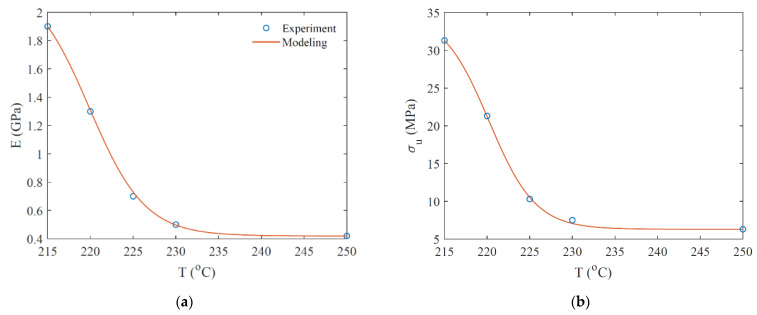
Young modulus (**a**) and ultimate strength (**b**) of PLA printed sample at different printing temperatures.

The results of the uniaxial tensile test for samples printed at different temperatures, with flow rates set in Figure 3a, are shown in Figure 5. In Figure 5a, the Young modulus presents a high variation in terms of printing temperature, dropping from 1.9 GPa to 0.4 GPa (79% reduction) in a 35 °C interval. Similarly, the ultimate strength decreases from 31 MPa to 6 MPa (80% reduction). The variations in the Young modulus and ultima strength are modeled by Equation (1). The corresponding material properties can be found in Table 1.

**Figure 6:**
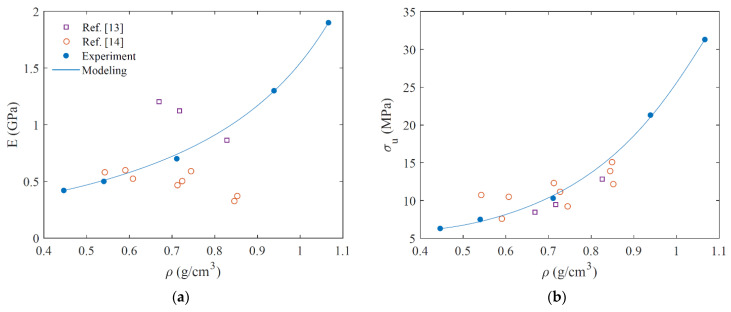
Young modulus (**a**) and ultimate strength (**b**) of 3D-printed foam material with different densities.

Figure 6 shows the tensile stiffness and strength as a function of density for the different printing temperatures, and benchmarks these results with syntactic 3D-printed foams. Comparing the results reported in the literature with those from the present method, it is seen that lower densities can be achieved at 0.4 g/cm^3^ by printing at 250 °C. It is also seen that the tensile stiffness and strength of the 3D-printed PLA foams in this research have an increasing trend with increases in density. A similar stiffness–density variation trend was reported for tensile strength in References [13,14], while a decreasing trend was observed for stiffness in those references.

## 4. Conclusions

This paper showed how FDM, when used as a 3D printing technology, can engineer lightweight PLA foams with a CFA. The density feature was experimentally investigated in terms of fabrication parameters such as printing temperature and flow rate, which affect the size of bubbles produced during fabrication. A set of parametric studies was carried out to examine the influence of printing temperature and flow rate on bubble size, micro-scale material connections, Young modulus, and strength. An interpolation function was introduced to accurately replicate the experimental data on flow rate, density and mechanical properties in terms of printing temperature. Finally, the following results can be concluded:

1. The filament is used directly in the manufacturing process, with no additive mixing during printing, which allows for a higher convenience in terms of usability;
2. The printed results do not contain foreign materials, unlike syntactic foams, which may increase recyclability;
3. The present method can improve the mechanical performance of previously researched 3D-printed foams;
4. The results present a higher strength and stiffness at higher densities compared with previously researched 3D-printed foams;
5. The material presents a high spectrum of properties, varying according to the printed temperature;
6. This wide range of properties could be leveraged in functionally graded prints for lightweight sandwich structures, presenting a potential alternative to ribs and spars that is easier to manufacture and faster to prototype.

## Figures and Tables

**Table 1:** The material constant parameters.

Parameters P(T)	$P_{l}$	$P_{h}$	$\gamma_{1}$	$\gamma_{2}$
Flow rate (%)	95	35	0.13	0.1315
Density (g/cm^3^)	1.07	0.44	0.15	0.1510
Young modulus (GPa)	1.9	0.42	0.15	0.1535
Ultimate strength (MPa)	25	6.3	0.18	0.1839

## Data Availability

Not applicable.
